# Supplementary material for: Global hypo-methylation in a proportion of glioblastoma enriched for an astrocytic signature is associated with increased invasion and altered immune landscape
Source: eLife. 2022 Nov 22;11:e77335. doi: 10.7554/eLife.77335 (PMC9681209; doi:10.7554/eLife.77335)
Supplement: Figure 2—source data 1. [file elife-77335-fig2-data1.zip › Figure_2_source_data_1/Figure_2B/homerResults/motif33.similar.html]

motif33

## Information for motif33

A
C
G
T
A
G
T
C
A
C
G
T
A
C
G
T
A
G
T
C
A
G
T
C
A
G
T
C
A
C
G
T
A
G
T
C
A
C
G
T
A
C
T
G
A
C
T
G
  
Reverse Opposite:  

A
G
T
C
A
G
T
C
C
G
T
A
A
C
T
G
C
G
T
A
A
C
T
G
A
C
T
G
A
C
T
G
C
G
T
A
C
G
T
A
A
C
T
G
C
G
T
A
  

|  |  |
| --- | --- |
| p-value: | 1e-42 |
| log p-value: | -9.892e+01 |
| Information Content per bp: | 1.530 |
| Number of Target Sequences with motif | 89.0 |
| Percentage of Target Sequences with motif | 3.85% |
| Number of Background Sequences with motif | 2.9 |
| Percentage of Background Sequences with motif | 0.81% |
| Average Position of motif in Targets | 90.3 +/- 49.1bp |
| Average Position of motif in Background | 118.7 +/- 48.3bp |
| Strand Bias (log2 ratio + to - strand density) | 0.4 |
| Multiplicity (# of sites on avg that occur together) | 1.03 |
| Motif File: | file (matrix) reverse opposite |

### Similar de novo motifs found

|  |  |  |  |  |  |  |  |
| --- | --- | --- | --- | --- | --- | --- | --- |
| Rank | Match Score | Redundant Motif | P-value | log P-value | % of Targets | % of Background | Motif file |
| 1 | 0.718 | T A G C T C G A T C G A C T G A C T A G C T A G T C A G T C G A C T G A T A C G C T G A T C A G C T G A C T A G C T A G | 1e-30 | -69.181122 | 3.16% | 0.71% | motif file (matrix) |
| 2 | 0.669 | A G T C A G T C A C G T A C G T A G T C A G T C A G T C C G T A A G T C A C G T | 1e-28 | -65.700800 | 3.07% | 0.79% | motif file (matrix) |
